# Dosimetric impact of Ac-227 in accelerator-produced Ac-225 for alpha-emitter radiopharmaceutical therapy of patients with hematological malignancies: a pharmacokinetic modeling analysis

**DOI:** 10.1186/s40658-021-00410-6

**Published:** 2021-08-18

**Authors:** George Sgouros, Bin He, Nitya Ray, Dale L. Ludwig, Eric C. Frey

**Affiliations:** 1 Russell H. Morgan Department of Radiology and Radiological Science, School of Medicine, Johns Hopkins University, CRB II 4M.61, 1550 Orleans St., Baltimore, MD 21287 USA; 2 Radiopharmaceutical Imaging and Dosimetry, LLC (Rapid), Baltimore, MD USA; 3 Actinium Pharmaceuticals, Inc., New York, NY USA

**Keywords:** Actinium-225, Alpha-emitter, Radiopharmaceutical therapy, Dosimetry, Compartmental modeling

## Abstract

**Background:**

Actinium-225 is an alpha-particle emitter under investigation for use in radiopharmaceutical therapy. To address limited supply, accelerator-produced ^225^Ac has been recently made available. Accelerator-produced ^225^Ac via ^232^Th irradiation (denoted ^225/7^Ac) contains a low percentage (0.1–0.3%) of ^227^Ac (21.77-year half-life) activity at end of bombardment. Using pharmacokinetic modeling, we have examined the dosimetric impact of ^227^Ac on the use of accelerator-produced ^225^Ac for radiopharmaceutical therapy. We examine the contribution of ^227^Ac and its daughters to tissue absorbed doses. The dosimetric analysis was performed for antibody-conjugated ^225/7^Ac administered intravenously to treat patients with hematological cancers. Published pharmacokinetic models are used to obtain the distribution of ^225/7^Ac-labeled antibody and also the distribution of either free or antibody-conjugated ^227^Th.

**Results:**

Based on our modeling, the tissue specific absorbed dose from ^227^Ac would be negligible in the context of therapy, less than 0.02 mGy/MBq for the top 6 highest absorbed tissues and less than 0.007 mGy/MBq for all other tissues. Compared to that from ^225^Ac, the absorbed dose from ^227^Ac makes up a very small component (less than 0.04%) of the total absorbed dose delivered to the 6 highest dose tissues: red marrow, spleen, endosteal cells, liver, lungs and kidneys when accelerator produced ^225/7^Ac-conjugated anti-CD33 antibody is used to treat leukemia patients. For all tissues, the dominant contributor to the absorbed dose arising from the ^227^Ac is ^227^Th, the first daughter of ^227^Ac which has the potential to deliver absorbed dose both while it is antibody-bound and while it is free. CONCLUSIONS: These results suggest that the absorbed dose arising from ^227^Ac to normal organs would be negligible for an ^225/7^Ac-labeled antibody that targets hematological cancer.

## Introduction

Alpha-particle emitter radiopharmaceutical therapy (αRPT) is a promising new approach to cancer therapy. It has been found impervious to conventional resistance mechanism that make traditional therapy ineffective [1]. Encouraging results have been observed in early clinical studies utilizing ^225^Ac to deliver alpha-particles for both hematologic and solid tumor treatment, including programs targeting CD33 in acute myeloid leukemia and PSMA in castrate-resistant prostate cancer [2, 3]. However, current limitation of available ^225^Ac supply, due to the fixed output from ^229^Th generator, has been a concern that has impacted preclinical and clinical use of ^225^Ac-based αRPT [4]. Accordingly, a number of alternative production methods have been examined as potential sources of large and sustainable quantities of ^225^Ac [5–7]. Accelerator-produced ^225^Ac via ^232^Th irradiation (hereafter denoted as ^225/7^Ac) contains 0.1 to 0.3% ^227^Ac (21.77-year half-life) activity at end of bombardment [8]. To account for the time elapsed for processing, transport and injectate preparation, we consider a scenario where the injected, ^225/7^Ac radiolabeled conjugate contains 0.7% ^227^Ac.

**Fig. 1:**
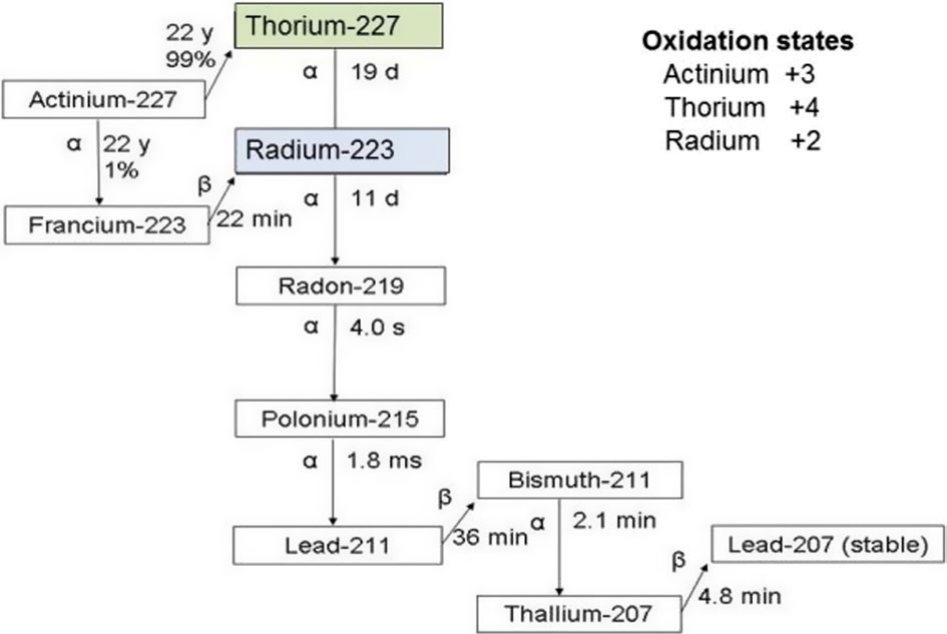
Actinium-227 decay scheme [28]

Actinium-227 decays by beta-particle emission primarily (99%) to thorium-227 (^227^Th; 18.68-d half-life) which in turn decays to radium-223 (^223^Ra, 11.43-d half-life) and a series of other alpha- and beta-emitting daughters to stable lead-207 (^207^Pb) (Fig. 1).

Using pharmacokinetic modeling, this work examines the contribution of ^227^Ac and its daughters to tissue absorbed doses when ^225/7^Ac-labeled antibody is administered intravenously to treat patients with hematological cancers (e.g., acute myeloid leukemia (AML) and/or myelodysplastic syndrome (MDS)).

## Methods

### Overview

Published pharmacokinetic models are used to obtain the distribution of ^225/7^Ac-labeled antibody and also the distribution of either free or antibody-conjugated ^227^Th. Since ^227^Th is obtained from the beta decay branch (99% yield) of ^227^Ac rather than a more energetically disruptive alpha-emitter decay, as has been observed with the ^212^Pb/^212^Bi delivery for α-emitter radiopharmaceutical therapy, [9–12], it is likely that a significant fraction of the ^227^Th generated remains antibody-conjugated. A pharmacokinetic model representing the distribution of radiolabeled antibody in patients with hematologically distributed cancer is adapted from reference [13] to obtain the pharmacokinetics for ^225/7^Ac and ^227^Th-labeled antibody. A model representing the pharmacokinetics of free ^227^Th is used to model the distribution of unconjugated ^227^Th [14]. Under both circumstances, ^223^Ra generated by ^227^Th decay is simulated using a pharmacokinetic model that is relevant to free ^223^Ra [15]. The 1% of ^227^Ac that decays to francium-223 (^223^Fr, T ½ = 22 min) is considered to have a negligible impact on tissue absorbed dose relative to that from ^227^Th which is already expected to be very low because of the low initial amount of ^227^Ac in ^225/7^Ac. Calculations were performed assuming 1 kg (10^12^ antigen-positive cells) in an adult female. The individual model simulations (i.e., Ab model, and the free ^227^Th and ^223^Ra models) are not coupled to each other. Rather, the biodistribution of free ^227^Th or ^223^Ra generated in the course of the simulation is assumed distributed throughout the body as it is created according to the kinetics described by the corresponding model (see Eqs. 17 and 18 of the “Appendix”).

### Biokinetic modeling

Table 1 summarizes the various models that were used to simulate the pharmacokinetics (PK) of each radionuclide. All compartmental models were solved using the simulation analysis and modeling software package (SAAM II, The Epsilon Group, Charlottesville, VA). Detailed model equations are listed in the “Appendix”.

**Table 1:** PK models used for each radionuclide

Radionuclide	Model used
Ac-225	Ab PK model, (Fig. 2)
Ac-227	Ab PK model, (Fig. 2)
Th-227	f_Ab_: Ab PK model (Fig. 2)
	1-f_Ab_: Free thorium model (Fig. 3)
Ra-223 and all its daughters	Free radium model (Fig. 4)

#### Antibody pharmacokinetic modeling

**Fig. 2:**
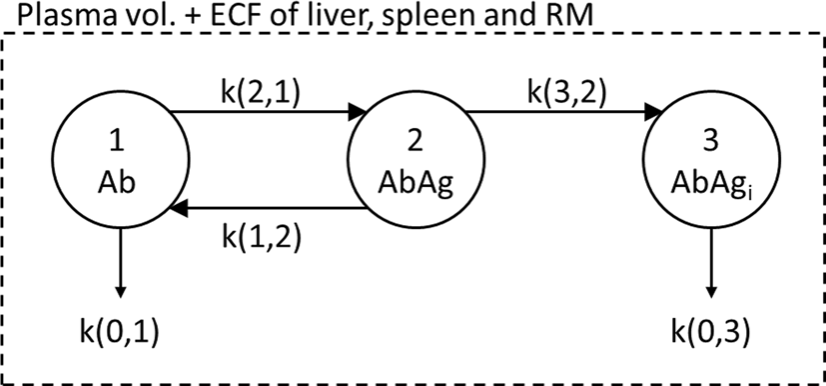
Pharmacokinetic model for radiolabeled antibody. The dotted line corresponds to the distribution volume of IV-administered antibody; ECF = extracellular fluid volume. Compartments 1 and 2 represent the free, (Ab) and antigen-bound antibody (AbAg) states, respectively. Compartment 3 represents internalized AbAg. The figure is adapted from reference [13]

The radiolabeled antibody model is depicted in Fig. 2. This model is used to derive the kinetics of antibody-bound radionuclides. Radiolabeled antibody (Ab) is administered in the vascular space (compartment 1). It binds to antigen sites via saturable (non-linear) binding represented by the rate parameter k(2,1), which is a function of the affinity constant and the number of free antigen sites available (see equations in the “Appendix”). Antigen-bound antibody (AbAg) in compartment 2 may dissociate to return to the free radiolabeled antibody state by a rate constant, k(1,2) that is equal to the dissociation rate of antigen-bound antibody; AbAg may also internalize via k(3,2) into an intracellular compartment (compartment 3) where it is no longer available for dissociation but is cleared via catabolism at a rate represented by k(0,3). This lumped parameter model neglects aspects related to spatial gradients and transport across vasculature and is, therefore, specific to a radiopharmaceutical therapy of rapidly accessible antigen-positive cells within a vascular space that includes the plasma volume and the extracellular fluid (ECF) volume of the liver, spleen and red marrow (represented by the dotted box). The essential components of this model have been previously validated using patient data [13].

#### Thorium biokinetic modeling

**Fig. 3:**
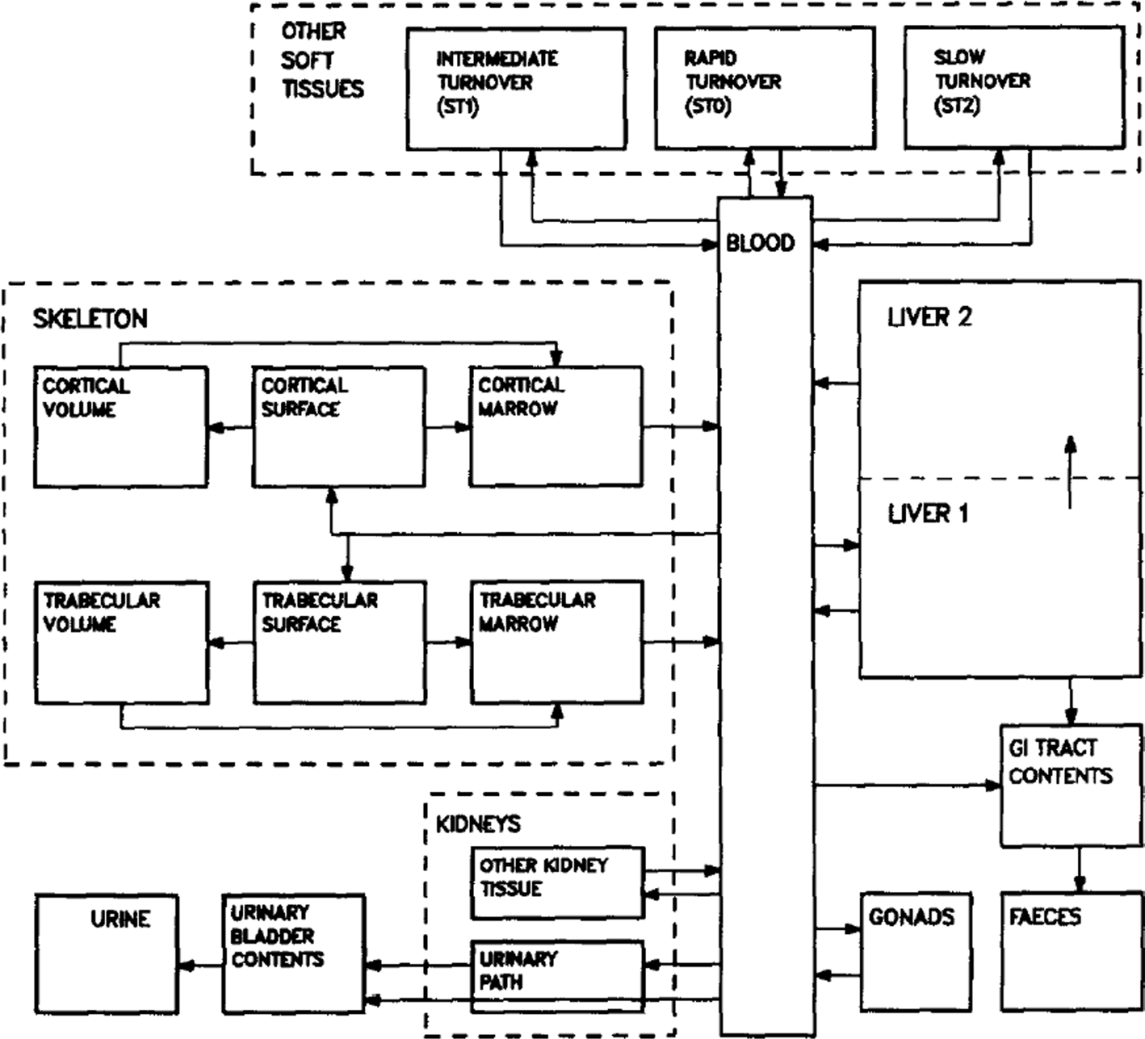
ICRP biokinetic model for thorium. Details and parameter values are provided in reference [14]

Time–activity curves for the fraction of ^227^Th that is not antibody-bound following the decay of ^227^Ac are given by the biokinetic model shown in Fig. 3. This model was developed and has been validated by Committee 2 of the International Commission on Radiological Protection (ICRP) [14, 16].

#### Radium biokinetic modeling

**Fig. 4:**
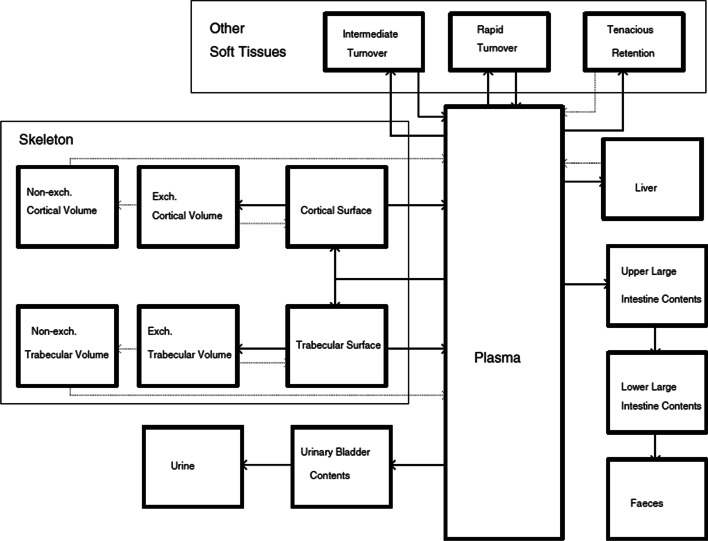
Biokinetic model for radium as represented in reference [15]. The solid arrows are relevant to the biokinetics of ^223^Ra

The biokinetic model for free radium is depicted in Fig. 4. It is based on an ICRP model describing the behavior of alkaline earth elements (“Appendix” of reference [17]), as implemented by Lassmann et al. to calculate normal tissue dosimetry for ^223^RaCl_2_ [15].

### Time-integrated activity coefficients (TIACs)

The time-integrated activity (TIA), for each source region, $r_{i}$, ($\tilde{A}\left( {r_{i} } \right)$) was obtained by integrating model-derived pharmacokinetic data. The TIAC is given by dividing TIA by the administered activity of ^225^Ac or by expressing the pharmacokinetics as a fraction of the administered activity. Equations 4–7 in the “Appendix” were integrated numerically with the substitutions indicated in equations: 8–10; 11–13, and 14–16, to get TIAC for ^225^Ac, ^227^Ac, and the antibody-bound fraction of ^227^Th, respectively. The TIAC for free ^227^Th and ^223^Ra was obtained by numerically integrating Eqs. 17 and 18. Numerical integration was performed using the trapezoidal method.

#### TIAC apportionment

Model-derived TIAC was apportioned to tissue parenchyma as specified by the pharmacokinetic models. TIAC calculated for blood (central compartment) was apportioned to all tissue according to their blood volume [18]. The daughters of ^225^Ac up to ^213^Bi, respectively, were assumed to decay at the site of ^225^Ac decay. Likewise the daughters of ^213^Bi were assumed to decay at the site of ^213^Bi decay. The TIAC, in each case was adjusted by the net yield of each daughter relative to the corresponding parent.

The same approach was taken for the daughters of ^223^Ra.

#### Absorbed dose calculations

Absorbed dose calculations were performed using the MIRD Committee S-value based method as described in pamphlet 21 [19]. The International Commission on Radiological Protection (ICRP) recently released absorbed fractions for a new series of phantoms that include far more tissues than were previously available [20]. The new absorbed fractions handle electron emissions far better than prior absorbed fractions which assumed that all or none of the energy associated with electron emissions was absorbed in tissues; absorption of alpha-particle energy is also appropriately considered [18]. A detailed comparison of the results obtained using OLINDA [21] and the new set of ICRP data has been published [22]. The calculations were performed using, newly developed software package, 3D-RD-S (Radiopharmaceutical Imaging and Dosimetry, LLC (Rapid), Baltimore MD), designed to account for the complexity of alpha-particle emitter dosimetry, in particular the differential fate of alpha-particle emitting daughters [23]. Absorbed doses from alpha-particles would ordinarily be multiplied by an RBE value of 5 [24, 25]. We have chosen not to use this factor and rather report the absorbed dose for each emission type directly. This approach provides all the information needed to apply an RBE value to the alpha-component of the absorbed dose.

#### Radionuclide decay scheme data

Decay schemes and half-lives for ^225^Ac and ^227^Ac and their daughters were obtained from ICRP publication 107 [26].

#### PK model parameter values

Table 2 lists the parameter values for the antibody PK model, and the free ^227^Th model and the ^223^Ra model parameters are available in the publications related to the models that are cited above.

**Table 2:** Model parameter values

Parameter	Value	References
$Ag_{0}$ (nmol)	14.95	Assuming 90% of 1 kg (10^12^) antigen-positie cells are in the distribution volume, $V_{d}$
$$V_{d}$$ (L)	3.8	[13]
$k_{ + }$ (M^−1^ h^−1^)	0.5	[13]
$k_{ - }$ (h^−1^)	0.003	[13]
$$T_{c}$$ (h)	40	[13]
$T_{i}$ (h)	0.5	Estimated
$T_{ci}$ (h)	100	Estimated
$f_{L1} ,f_{S1}$	0.18, 0.12	[13]
$f_{L2} ,f_{S2}$	0.08, 0.06	[13]
$V_{RMECF} ,V_{d}$ (L)	0.22, 3.8	[13]
$f_{Ac227}$	0.007	Based on EOB fraction and assuming two ^225^Ac half-lives until time to injection
$f_{Ab}$	0.7	Estimated

## Results

### Ab–Ag pharmacokinetic model

**Fig. 5:**
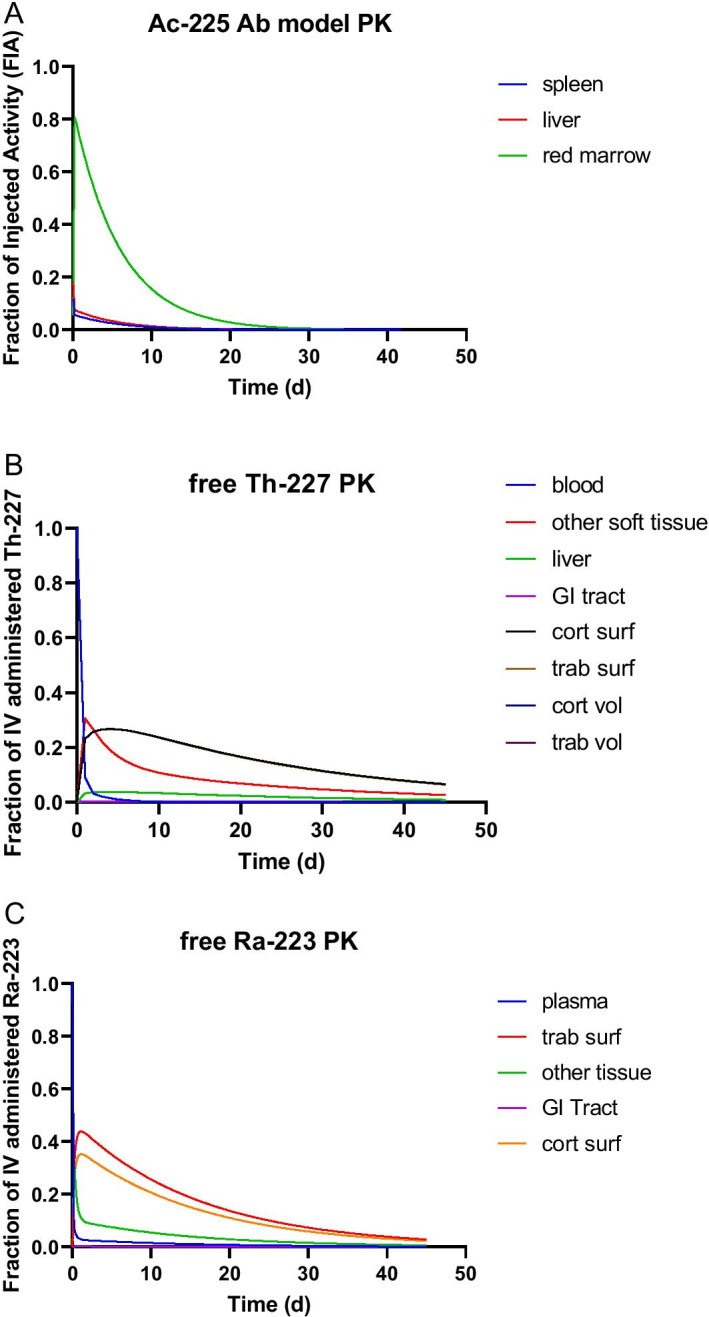
Results of **A** Ab PK model simulations for ^225^Ac-bound Ab. **B** PK for free ^227^Th **C** PK for free ^223^Ra

The time–activity data obtained from Ab PK model simulations are plotted in Fig. 5A.

The PK for ^227^Ac-bound Ab is identical to that shown in Fig. 5, except that all data are scaled by 0.07% (= $f_{Ac227}$, Table 2).

Since 99% of ^227^Ac decays by beta-particle emission, which is less energetically disruptive than alpha-particle decay, the assumption is made that 70% (= $f_{Ab}$) of the daughter radionuclide, ^227^Th, remains antibody-bound and obeys PK that is identical to that shown in Fig. 5A, except that all data are scaled by $f_{Ac227} \cdot f_{Ab}$. The remaining 30% is assumed to obey the pharmacokinetics of free ^227^Th (Fig. 5B); this value was chosen as it is consistent with the retention of ^212^Bi following decay of ^212^Pb, also a beta-decay transition [27]. Since ^227^Th decays by alpha-particle emission to ^223^Ra, all of the ^223^Ra generated, regardless of whether the ^227^Th was Ab-bound or free is assumed to follow free radium kinetics (Fig. 5C).

As indicated in the text, Fig. 5A is scaled relative to an arbitrary amount of administered ^225/7^Ac that is antibody-bound. In other words 1 MBq of ^225/7^Ac-Ab administered should be multiplied by the fraction of injected activity (FIA) values indicated on the y-axis to obtain the corresponding amount of ^227^Ac or ^227^Th activity. The PK data plotted on Fig. 5B, C should be multiplied by $f_{Ac227} \cdot (1 - f_{Ab} )$ and $f_{Ac227}$, respectively, to convert the results to per MBq of ^22/75^Ac administered.

The resulting time–activity curves for each “species” were numerically integrated to obtain the TIAC for each of the indicated tissues (Table 3).

**Table 3:** Summary of model-derived TIAC for each radionuclide

Tissue	Radionuclide TIAC (MBq-h/MBq ^225/7^Ac administered)				
	^225^Ac-Ab, ^221^Fr, ^217^At	Free ^213^Bi	^227^Ac-Ab	Total ^227^Th	^223^Ra + daughters
Adipose tissue	1.881E−01	9.406E−02	9.287E−06	3.893E−04	1.488E−04
Adrenals	1.440E−03	7.198E−04	7.106E−08	7.147E−08	2.430E−08
Lungs	2.999E−01	1.500E−01	1.480E−05	1.489E−05	5.063E−06
Brain	2.879E−02	1.440E−02	1.421E−06	1.429E−06	4.860E−07
Breasts	1.384E−02	6.919E−03	6.831E−07	6.870E−07	2.336E−07
Cortical bone surface	0.000E+00	0.000E+00	0.000E+00	7.814E−04	5.287E−04
Cortical bone	1.919E−02	9.597E−03	9.475E−07	1.333E−06	3.240E−07
Heart wall	2.399E−02	1.200E−02	1.184E−06	1.191E−06	4.050E−07
Kidneys	4.799E−02	0.000E+00	2.369E−06	5.800E−05	8.100E−07
Left colon contents	0.000E+00	0.000E+00	0.000E+00	3.792E−06	1.198E−06
Left colon wall	2.126E−02	1.063E−02	1.050E−06	9.123E−06	2.719E−06
Liver	8.255E+00	2.064E+00	5.709E−04	3.393E−04	4.050E−06
Muscle	2.519E−01	1.260E−01	1.244E−05	1.251E−05	4.253E−06
Ovaries	4.799E−04	2.399E−04	2.369E−08	7.925E−07	8.100E−09
Pancreas	1.440E−02	7.198E−03	7.106E−07	7.147E−07	2.430E−07
RM	8.396E+01	8.185E+00	5.902E−03	2.368E−03	1.620E−06
Right colon contents	0.000E+00	0.000E+00	0.000E+00	5.807E−06	1.834E−06
Right colon wall	2.126E−02	1.063E−02	1.050E−06	7.107E−06	2.082E−06
Rectosigmoid colon contents	0.000E+00	0.000E+00	0.000E+00	5.916E−06	1.869E−06
Rectosigmoid colon wall	1.026E−02	5.132E−03	5.067E−07	6.732E−06	2.048E−06
Spleen	6.153E+00	4.471E−01	4.263E−04	3.367E−04	5.670E−07
Trabecular bone surface	0.000E+00	0.000E+00	0.000E+00	7.688E−04	6.575E−04
Trabecular bone	2.879E−02	1.440E−02	1.421E−06	5.775E−06	4.860E−07
Thymus	5.535E−04	2.768E−04	2.732E−08	2.748E−08	9.343E−09
Thyroid	1.440E−03	7.198E−04	7.106E−08	7.147E−08	2.430E−08
Urinary bladder content	0.000E+00	0.000E+00	0.000E+00	1.669E−04	0.000E+00
Uterus	2.214E−03	1.107E−03	1.093E−07	1.099E−07	3.737E−08

### Absorbed doses

Table 4 lists absorbed doses for ^225^Ac and ^227^Ac; the absorbed dose from each particle type is provided separately. Table 5 lists the absorbed dose to selected tissues from ^225^Ac, ^227^Ac and their respective daughters. (Contributions from the 1% decay of ^227^Ac to ^223^Fr and daughters with a yield of less than 10^–4^% are not included.)

**Table 4:** ^225^Ac (including daughters) and ^227^Ac (including daughters) absorbed doses for selected tissues^1^

	Absorbed dose (mGy/1 MBq ^225/7^Ac administered); RBE not applied									
	^225^Ac + daughters					^227^Ac + daughters				
	Alpha	Beta	Electron	Photon	Total	Alpha	Beta	Electron	Photon	Total
Red marrow	5.40E+02	1.32E+00	1.23E+00	1.28E−01	5.43E+02	6.66E−03	1.09E−04	1.05E−04	1.62E−05	6.89E−03
Spleen	3.74E+02	8.37E−01	6.64E−01	1.62E−01	3.76E+02	6.23E−03	2.06E−05	8.56E−05	2.17E−05	6.36E−03
Endosteal bone surface	2.48E+02	5.75E−01	4.19E−01	5.53E−02	2.49E+02	1.81E−02	1.26E−04	9.30E−05	4.95E−05	1.84E−02
Liver	5.59E+01	4.13E−01	1.02E−01	7.61E−02	5.65E+01	6.82E−04	4.05E−06	9.34E−06	6.98E−06	7.03E−04
Lungs	4.48E+00	7.14E−02	9.08E−03	5.22E−02	4.61E+00	1.42E−04	4.22E−06	1.16E−06	5.52E−06	1.53E−04
Kidneys	1.49E+00	2.61E−03	2.71E−03	5.42E−02	1.54E+00	5.86E−04	1.38E−06	7.21E−06	7.44E−06	6.02E−04
Pancreas	1.34E+00	1.84E−02	2.53E−03	5.12E−02	1.41E+00	4.25E−05	9.62E−07	3.31E−07	5.56E−06	4.94E−05
Adrenals	1.25E+00	2.83E−02	2.77E−03	9.02E−02	1.37E+00	3.98E−05	1.64E−06	3.59E−07	9.31E−06	5.11E−05
Heart wall	1.11E+00	1.89E−02	2.27E−03	4.90E−02	1.18E+00	3.52E−05	9.72E−07	2.86E−07	4.83E−06	4.13E−05
Thyroid	9.95E−01	1.25E−02	1.84E−03	3.60E−02	1.05E+00	3.16E−05	7.87E−07	2.46E−07	4.45E−06	3.71E−05
Ovaries	5.46E−01	7.01E−03	1.02E−03	3.75E−02	5.91E−01	2.38E−04	7.09E−07	2.95E−06	6.00E−06	2.47E−04
Thymus	3.61E−01	1.61E−02	1.29E−03	5.32E−02	4.32E−01	1.15E−05	1.87E−06	1.59E−07	5.70E−06	1.92E−05
Uterus	3.61E−01	4.94E−03	6.80E−04	3.47E−02	4.02E−01	1.15E−05	5.15E−07	1.03E−07	6.12E−06	1.82E−05
Breast	3.61E−01	4.77E−03	6.74E−04	2.02E−02	3.87E−01	1.15E−05	3.11E−07	8.85E−08	2.06E−06	1.39E−05
Brain	2.87E−01	6.25E−03	6.61E−04	1.76E−02	3.12E−01	9.12E−06	2.78E−06	1.40E−07	4.70E−06	1.67E−05
Muscle	1.89E−01	6.02E−03	5.51E−04	2.05E−02	2.16E−01	6.00E−06	1.63E−06	9.57E−08	3.26E−06	1.10E−05
Salivary glands	0.00E+00	1.03E−03	4.50E−05	2.22E−02	2.32E−02	0.00E+00	1.99E−06	5.18E−08	4.49E−06	6.54E−06
Eye lens	0.00E+00	2.49E−05	1.65E−07	1.10E−02	1.10E−02	0.00E+00	6.06E−08	5.69E−10	2.95E−06	3.01E−06

^1^Assuming 0.7% ^227^Ac in accelerator produced ^225/7^Ac and that 70% of the ^227^Th produced by beta decay of ^227^Ac-labeled antibody

remains attached to the antibody

**Table 5:** Absorbed dose delivered by each daughter* for selected tissues

	Absorbed Dose (mGy/1 MBq ^225/7^Ac Administered); RBEnot applied															
	^225^Ac	^221^Fr	^217^At	^213^Bi	^213^Po	^209^Pb	^209^Tl	^227^Ac	^227^Th	^223^Ra	^219^Rn	^215^Po	^211^Pb	^211^Bi	^207^Tl	^211^Po
Red marrow	1.61E+02	1.71E+02	1.88E+02	1.23E+00	2.09E+01	4.50E−01	3.22E−02	1.87E−04	5.06E−03	3.38E−04	3.99E−04	4.37E−04	3.51E−05	3.86E−04	3.76E−05	1.21E−06
Spleen	1.10E+02	1.20E+02	1.34E+02	7.67E−01	1.13E+01	2.66E−01	2.43E−02	1.10E−04	6.19E−03	1.05E−05	1.20E−05	1.29E−05	1.01E−06	1.16E−05	9.00E−07	3.59E−08
Endosteal bone surface	7.41E+01	7.89E+01	8.61E+01	5.56E−01	9.30E+00	1.85E−01	1.47E−02	7.77E−05	5.63E−03	2.81E−03	3.24E−03	3.34E−03	6.05E−05	3.18E−03	5.84E−05	9.24E−06
Liver	1.53E+01	1.66E+01	1.86E+01	3.88E−01	5.39E+00	1.29E−01	1.51E−02	1.53E−05	6.50E−04	7.86E−06	8.91E−06	9.53E−06	8.64E−07	8.61E−06	7.46E−07	2.66E−08
Lungs	1.13E+00	1.24E+00	1.36E+00	7.45E−02	7.88E−01	2.03E−02	5.32E−03	8.35E−07	6.10E−05	1.92E−05	2.22E−05	2.40E−05	2.17E−06	2.15E−05	2.13E−06	6.67E−08
Kidneys	4.64E−01	5.14E−01	5.48E−01	1.44E−02	3.34E−06	3.59E−04	3.92E−03	3.37E−07	5.64E−04	8.11E−06	9.09E−06	9.67E−06	8.81E−07	8.78E−06	6.97E−07	2.70E−08
Pancreas	3.46E−01	3.85E−01	4.06E−01	2.89E−02	2.35E−01	5.69E−03	4.32E−03	2.46E−07	2.13E−05	6.13E−06	6.78E−06	7.16E−06	6.83E−07	6.54E−06	4.89E−07	2.00E−08
Adrenals	3.33E−01	3.80E−01	3.80E−01	4.63E−02	2.20E−01	6.86E−03	7.50E−03	2.56E−07	2.33E−05	6.04E−06	6.47E−06	6.70E−06	1.11E−06	6.23E−06	8.51E−07	1.87E−08
Heart wall	2.89E−01	3.22E−01	3.36E−01	2.77E−02	1.95E−01	5.19E−03	3.96E−03	2.18E−07	1.78E−05	5.08E−06	5.62E−06	5.93E−06	6.41E−07	5.42E−06	4.92E−07	1.66E−08
Thyroid	2.57E−01	2.87E−01	3.02E−01	1.88E−02	1.75E−01	4.06E−03	2.68E−03	1.83E−07	1.56E−05	4.81E−06	5.14E−06	5.32E−06	6.59E−07	4.95E−06	4.07E−07	1.49E−08
Ovaries	1.45E−01	1.66E−01	1.66E−01	1.37E−02	9.59E−02	2.25E−03	2.46E−03	1.03E−07	2.35E−04	3.00E−06	2.96E−06	2.92E−06	6.22E−07	2.82E−06	3.77E−07	8.21E−09
Thymus	1.04E−01	1.22E−01	1.10E−01	2.49E−02	6.35E−02	2.97E−03	4.09E−03	1.12E−07	8.70E−06	2.21E−06	2.06E−06	1.93E−06	1.15E−06	1.95E−06	9.85E−07	5.48E−09
Uterus	9.85E−02	1.15E−01	1.10E−01	1.14E−02	6.35E−02	1.53E−03	2.28E−03	7.00E−08	9.24E−06	2.16E−06	2.03E−06	1.93E−06	5.15E−07	1.93E−06	2.77E−07	5.47E−09
Breast	9.43E−02	1.07E−01	1.10E−01	9.37E−03	6.35E−02	1.51E−03	1.79E−03	6.85E−08	6.10E−06	1.72E−06	1.87E−06	1.93E−06	2.56E−07	1.79E−06	1.62E−07	5.41E−09
Brain	7.58E−02	8.61E−02	8.71E−02	9.24E−03	5.05E−02	1.51E−03	1.41E−03	5.68E−08	5.96E−06	2.29E−06	1.86E−06	1.54E−06	1.76E−06	1.73E−06	1.51E−06	4.43E−09
Muscle	5.22E−02	6.08E−02	5.74E−02	9.60E−03	3.32E−02	1.27E−03	1.61E−03	4.26E−08	4.48E−06	1.33E−06	1.15E−06	1.01E−06	9.87E−07	1.08E−06	8.74E−07	2.89E−09
Salivary glands	5.12E−03	1.06E−02	8.96E−05	5.67E−03	1.36E−06	1.13E−04	1.61E−03	4.54E−09	2.44E−06	9.21E−07	3.92E−07	1.19E−09	1.34E−06	3.21E−07	1.09E−06	1.47E−10
Eye lens	2.34E−03	5.22E−03	4.54E−05	2.56E−03	7.45E−07	1.02E−06	8.29E−04	1.63E−09	1.45E−06	6.46E−07	2.88E−07	9.11E−10	3.42E−07	2.34E−07	4.39E−08	1.08E−10

^*^Contributions from the 1% decay of ^227^Ac to ^223^Fr and daughters with a yield of less than 10^–4^% are not included

**Fig. 6:**
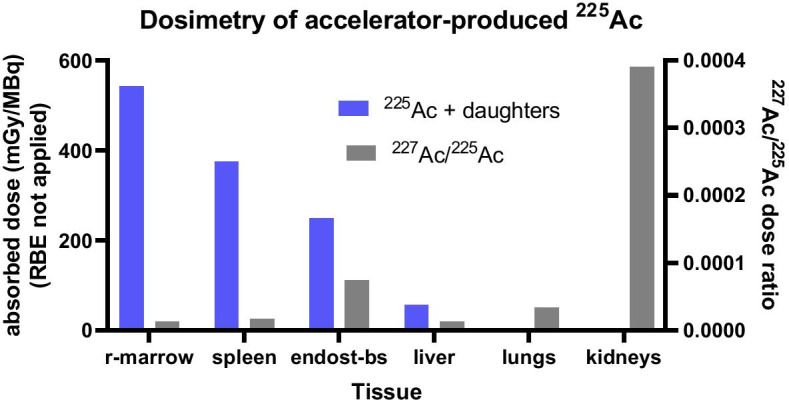
Total absorbed dose to tissue from 1 MBq administration of ^225/7^Ac-labeled anti-CD33 antibody in patients with leukemia. The left axis gives the absorbed dose (blue bars) from ^225^Ac and all daughters. The right axis is the ^227^Ac to ^225^Ac absorbed dose (including daughters) ratio (grey bars)

The absorbed dose from ^225^Ac and its daughters, along with the ^227^Ac to ^225^Ac absorbed dose ratio for the top 6 tissues by total absorbed dose, is depicted in Fig. 6.

## Discussion

The alpha emitter ^225^Ac is a promising radionuclide for the generation of potent radiopharmaceutical agents for hematologic and solid tumor malignancies. However, commercial-scale supply concerns regarding purified ^225^Ac have limited more widespread clinical research and development of ^225^Ac-based αRPT. As a result, a number of alternative production methods have been examined as potential sources of large and scalable quantities of ^225^Ac, including accelerator-produced ^225^Ac via ^232^Th irradiation. However, accelerator-produced ^225^Ac contains ^227^Ac as an impurity in the purified material. We undertook this work to investigate the dosimetric impact of the ^227^Ac present in accelerator-produced ^225^Ac used for αRPT therapy in hematologic malignancies. Our modeling results determined that the tissue absorbed dose from ^227^Ac would be negligible in the context of therapy, less than 0.02 mGy/MBq for the top 6 highest absorbed dose tissues and less than 0.007 mGy/MBq for all other tissues. Compared to that from ^225^Ac, the absorbed dose from ^227^Ac would make up a very small component (< 0.04%) of the total absorbed dose delivered to the 6 highest dose tissues: red marrow, spleen, endosteal cells, liver, lungs and kidneys when accelerator produced ^225/7^Ac-conjugated anti-CD33 antibody would be used to treat leukemia patients. For all tissues evaluated, the dominant contributor to the absorbed dose arising from the ^227^Ac is ^227^Th, the first daughter of ^227^Ac, which has the potential to deliver absorbed dose both while it is antibody-bound and while it is free. These results suggest that the absorbed dose arising from ^227^Ac to normal organs would be negligible for an ^225/7^Ac-labeled antibody that targets hematological cancer.

In addition to the models used in these simulations and their related parameters, the following series of assumptions were used to arrive at these conclusion: (1) At time of administration there is 0.7% ^227^Ac in the ^225/7^Ac-conjugated anti-CD33 antibody. (2) 70% of the ^227^Th resulting from ^227^Ac decay remains antibody-bound and follows the same kinetics as the actinium-conjugated antibody. (3) ^227^Th decay releases free ^223^Ra. Under the simulation conditions described above, the spleen, red marrow, endosteal cells and liver would receive the highest absorbed doses from ^227^Ac and its daughters. The simulation also assumes high purity in the radiolabeled material so that loss of the labeled Ab also removes the Ac-227 conjugated to the Ab. It should be noted that different simulation models, parameter values and assumptions will give different results. In particular, these results may not apply to non-antibody carriers. The simulations and absorbed dose calculations were performed assuming 1 kg of antigen-positive cells in an adult female. Within the context of antibody-targeting of hematologic malignancies, alternative assumptions regarding percent ^227^Ac in the injectate and the fraction of ^227^Th that remains-antibody-bound may be implemented by scaling the listed absorbed dose values by the ratio of the new values with those used in this paper (e.g., by considering the scaling applied in Eqs. 8–16. For example, lower Ab retention of ^227^Th following decay of ^227^Ac may be obtained by scaling ^227^Th and daughter absorbed doses by the new retention fraction divided by 0.7 (= $f_{Ab}$). Such scaling can also account for injectate purity.

## Conclusions

Using a pharmacokinetic model relevant to treating patients with leukemia and models describing the PK of free thorium and radium, the dose contribution of a 0.7% ^227^Ac in accelerator-produced ^225^Ac would be negligible in the context of αRPT therapy, less than 0.02 mGy/MBq for the top 6 highest absorbed tissues and less than 0.007 mGy/MBq for all other tissues.

The conclusion above is specific to the parameter values and assumptions outlined and may not apply to lower molecular weight agents or other cancer targets.

## Data Availability

The paper describes a series of simulations, all information required to repeat the simulations is included in the manuscript.

## Appendix: Model equations

The model is described by the following differential equations:

1 $$\frac{dAb}{{dt}} = k\left( {1,2} \right)AbAg - k\left( {0,1} \right)Ab - k\left( {2,1} \right)Ab$$

2 $$\frac{dAbAg}{{dt}} = k\left( {2,1} \right)Ab - k\left( {1,2} \right)AbAg - k\left( {3,2} \right)AbAg$$

3 $$\frac{{dAbAg_{i} }}{dt} = k\left( {3,2} \right)AbAg - k\left( {0,3} \right)AbAg_{i}$$

with $k\left( {1,2} \right)$ = $k_{ - }$*,* the AbAg dissociation rate; $k\left( {2,1} \right) = \frac{{k_{ + } }}{{V_{d} }} \cdot \left( {Ag_{0} - AbAg} \right)$, the time-dependent Ab association rate to free antigen (Ag) sites; $k\left( {0,1} \right) = \frac{{{\text{ln}}\left( 2 \right)}}{{T_{c} }}$, the clearance rate of Ab; $k\left( {3,2} \right) = \frac{{{\text{ln}}\left( 2 \right)}}{{T_{i} }}$, the internalization rate of AbAg; $k\left( {0,3} \right) = \frac{{{\text{ln}}\left( 2 \right)}}{{T_{ci} }}$, loss/catabolism rate of AbAg_i_; $k_{ + }$, the Ab, Ag association rate; $V_{d}$, initial distribution volume of antibody; $Ag_{0}$, total number of antigen sites; $T_{c}$, Ab clearance half-time; $T_{i}$, AbAg internalization half-time; $T_{ci}$, AbAg loss or catabolism half-time of internalized AbAg.

Using this model, the amount of radiolabeled antibody in plasma ($Q_{P} \left( t \right)$), liver ($Q_{L} \left( t \right)$), spleen ($Q_{S} \left( t \right)$) and red marrow ($Q_{RM} \left( t \right)$) as a function of time, *t*, may be obtained using the equations below:

4 $$Q_{P} \left( t \right) = \left( {1 - \frac{{V_{RMECF} }}{{V_{d} }}} \right) \cdot Ab\left( t \right)$$

5 $$Q_{L} \left( t \right) = f_{L1} \cdot Ab\left( t \right) + f_{L2} \cdot \left( {AbAg\left( t \right) + AbAg_{int} \left( t \right)} \right)$$

6 $$Q_{S} \left( t \right) = f_{S1} \cdot Ab\left( t \right) + f_{S2} \cdot \left( {AbAg\left( t \right) + AbAg_{int} \left( t \right)} \right)$$

7 $$Q_{RM} \left( t \right) = \frac{{V_{RMECF} }}{{V_{d} }} \cdot Ab\left( t \right) + \left( {1 - f_{L2} - f_{s2} } \right) \cdot \left( {AbAg\left( t \right) + AbAg_{int} \left( t \right)} \right)$$

with $f_{L1} ,f_{S1}$, fraction of Ab in the vascular or extracellular fluid space of the liver (L), or spleen (S); $f_{L2} ,f_{S2}$, fraction of AbAg in the liver (L), or spleen (S); $V_{RMECF} ,V_{d}$, red marrow ECF volume and total Ab distribution volumes.

Further details regarding this model, including the derivation of Eqs. 1–3, are in reference [13].

The time–activity curves for ^225^Ac, in plasma, liver, spleen and red marrow are given by substituting for $Ab\left( t \right)$, $AbAg\left( t \right)$, and $AbAg_{int} \left( t \right)$ in Eqs. 4–7 as follows:

8 $$Ab\left( t \right)_{Ac225} = (1 - f_{Ac227} ) \cdot e^{{ - \lambda_{Ac225} \cdot t}} \cdot Ab\left( t \right)$$

9 $$AbAg\left( t \right)_{Ac225} = (1 - f_{Ac227} ) \cdot e^{{ - \lambda_{Ac225} \cdot t}} \cdot AbAg\left( t \right)$$

10 $$AbAg_{int} \left( t \right)_{Ac225} = (1 - f_{Ac227} ) \cdot e^{{ - \lambda_{Ac225} \cdot t}} \cdot AbAg_{int} \left( t \right)$$

with $f_{Ac227}$, fraction of total radioactivity arising from ^227^Ac; $\lambda_{Ac225}$, transformation rate (= $\frac{{{\text{ln}}\left( 2 \right)}}{{T_{Ac225} }}$) of ^225^Ac; $T_{Ac225}$, physical half-life of ^225^Ac.

Similar substitutions apply for ^227^Ac and ^227^Th, in plasma, liver, spleen and red marrow are given by substituting for $Ab\left( t \right)$, $AbAg\left( t \right)$, and $AbAg_{int} \left( t \right)$ in Eqs. 4–7 as follows:

11 $$Ab\left( t \right)_{Ac227} = f_{Ac227} \cdot e^{{ - \lambda_{Ac227} \cdot t}} \cdot Ab\left( t \right)$$

12 $$AbAg\left( t \right)_{Ac227} = f_{Ac227} \cdot e^{{ - \lambda_{Ac227} \cdot t}} \cdot AbAg\left( t \right)$$

13 $$AbAg_{int} \left( t \right)_{Ac227} = f_{Ac227} \cdot e^{{ - \lambda_{Ac227} \cdot t}} \cdot AbAg_{int} \left( t \right)$$

The corresponding equations for ^227^Th activity in plasma, liver, spleen and red marrow that remains antibody-bound are:

14 $$Ab\left( t \right)_{Th227} = f_{Ab} \cdot e^{{ - \lambda_{Th227} \cdot t}} \cdot Ab\left( t \right)_{Ac227}$$

15 $$AbAg\left( t \right)_{Th227} = f_{Ab} \cdot e^{{ - \lambda_{Th227} \cdot t}} \cdot AbAg\left( t \right)_{Ac227}$$

16 $$AbAg_{int} \left( t \right)_{Th227} = f_{Ab} \cdot e^{{ - \lambda_{Th227} \cdot t}} \cdot AbAg_{int} \left( t \right)_{Ac227}$$

with $f_{Ab}$, fraction of ^227^Th generated by the decay of ^227^Ac that remains Ab-bound; $\lambda_{Th227}$, transformation rate of ^227^Th (= $\frac{{{\text{ln}}\left( 2 \right)}}{{T_{Th227} }}$); $T_{Th227}$, physical half-life of ^227^Th.

The activity of free ^227^Th (per MBq ^225^Ac-Ab administered) as a function of time in tissue, $i$, ($a_{i} \left( t \right)_{Th227}$) is obtained as follows:

17 $$a_{i} \left( t \right)_{Th227} = f_{Ac227} \cdot (1 - f_{Ab} ) \cdot e^{{ - \lambda_{Th227} \cdot t}} \cdot q_{i} \left( t \right)_{thorium}$$

with $q_{i} \left( t \right)_{thorium}$, thorium content in tissue, $i$, at time, $t$ assuming one (arbitrary) unit of thorium is injected at $t = 0$.

The activity of free ^223^Ra (per MBq ^225/7^Ac-Ab administered) as a function of time in tissue, $i$, ($a_{i} \left( t \right)_{Ra223}$) is obtained assuming all daughters of radium remain at the site of parent decay:

18 $$a_{i} \left( t \right)_{Ra223} = f_{Ac227} \cdot e^{{ - \lambda_{Ra223} \cdot t}} \cdot q_{i} \left( t \right)_{radium}$$

with $\lambda_{Ra223}$, transformation rate of ^223^Ra (= $\frac{{{\text{ln}}\left( 2 \right)}}{{T_{Ra223} }}$); $T_{Ra223}$, physical half-life of ^223^Ra; $q_{i} \left( t \right)_{radium}$, radium content in tissue, $i$, at time, $t$ assuming one (arbitrary) unit of radium is injected at $t = 0$.

## References

[1] Sgouros G (2019). α-particle–emitter radiopharmaceutical therapy: resistance is futile. Cancer Res.

[2] Jurcic JG (2020). Targeted alpha-particle therapy for hematologic malignancies. Semin Nucl Med.

[3] Lawal IO, Bruchertseifer F, Vorster M, Morgenstern A, Sathekge MM (2020). Prostate-specific membrane antigen-targeted endoradiotherapy in metastatic prostate cancer. Curr Opin Urol.

[4] REPORT ON JOINT IAEA-JRC WORKSHOP “SUPPLY OF ACTINIUM-225, 2018; Vienna, Austria.

[5] John K (2019). US DOE tri-lab production effort to provide accelerator-produced 225Ac for radiotherapy: 2019 update. J Nucl Med.

[6] Hoehr C, Bénard F, Buckley K (2017). Medical isotope production at TRIUMF—from imaging to treatment. Phys Procedia.

[7] Walsh KM. Producing Radioisotopes for Medical Imaging and Disease Treatment. In: Collide BNLRHi, ed. 2017. https://www.bnl.gov/rhic/news2/news.asp?a=12043&t=today. Accessed 14 May 2020.

[8] Zewei J, Ekaterina R, Darrell RF, Ekaterina D (2018). In vivo evaluation of free and chelated accelerator-produced Actinium-225—radiation dosimetry and toxicity results. Curr Radiopharm.

[9] Stallons TAR, Saidi A, Tworowska I, Delpassand ES, Torgue JJ (2019). Preclinical investigation of Pb-212-DOTAMTATE for peptide receptor radionuclide therapy in a neuroendocrine tumor model. Mol Cancer Ther.

[10] Delpassand E, Tworowska I, Shanoon F (2019). First clinical experience using targeted alpha-emitter therapy with Pb-212-DOTAMTATE (AlphaMedix (TM)) in patients th SSTR(+) neuroendocrine tumors. J Nucl Med.

[11] Brechbiel MW (2008). Bifunctional chelates for metal nuclides. Q J Nucl Med Mol Imaging.

[12] Ruble G, Wu C, Squire RA, Ganswo OA, Strand M (1996). The use of 212Pb-labeled monoclonal antibody in the treatment of murine erythroleukemia. Int J Radiat Oncol Biol Phys.

[13] Sgouros G, Graham MC, Divgi CR, Larson SM, Scheinberg DA (1993). Modeling and dosimetry of monoclonal antibody M195 (anti-CD33) in acute myelogenous leukemia. J Nucl Med.

[14] Thorium. Annals of the ICRP. 1995;25:39–55.

[15] Lassmann M, Nosske D (2013). Dosimetry of 223Ra-chloride: dose to normal organs and tissues. Eur J Nucl Med Mol Imaging.

[16] Leggett RW (2001). Reliability of the ICRP's dose coefficients for members of the public. 1. Sources of uncertainty in the biokinetic models. Radiat Prot Dosim.

[17] ICRP. Publication 67, Age-dependent Doses to Members of the Public from Intake of Radionuclides: Part 2; Ingestion Dose Coefficients,: ICRP; 1993. 67. 7978694

[18] Bolch WE, Jokisch D, Zankl M (2016). ICRP Publication 133: The ICRP computational framework for internal dose assessment for reference adults: specific absorbed fractions. Ann ICRP.

[19] Bolch WE, Eckerman KF, Sgouros G, Thomas SR (2009). MIRD pamphlet No. 21: a generalized schema for radiopharmaceutical dosimetry—standardization of nomenclature. J Nucl Med.

[20] Menzel HG, Clement C, DeLuca P (2009). ICRP Publication 110. Realistic reference phantoms: an ICRP/ICRU joint effort. A report of adult reference computational phantoms. Ann ICRP.

[21] Stabin MG, Sparks RB, Crowe E (2005). OLINDA/EXM: the second-generation personal computer software for internal dose assessment in nuclear medicine. J Nucl Med.

[22] Josefsson A, Hobbs RF, Ranka S (2018). Comparative dosimetry for (68)Ga-DOTATATE: impact of using updated ICRP phantoms, S values, and tissue-weighting factors. J Nucl Med.

[23] Hamacher KA, Sgouros G (1999). A schema for estimating absorbed dose to organs following the administration of radionuclides with multiple unstable daughters: a matrix approach. Med Phys.

[24] Feinendegen LE, McClure JJ (1997). Meeting report—alpha-emitters for medical therapy—workshop of the United States department of energy—Denver, Colorado, May 30–31, 1996. Radiat Res.

[25] Sgouros G, Allen BJ, Back T, et al. MIRD monograph: radiobiology and dosimetry for radiopahrmaceutical therapy with alpha-particle emitters. Sgouros G (editor). Reston VA: SNMMI; 2015.

[26] Eckerman K, Endo A (2008). ICRP Publication 107. Nuclear decay data for dosimetric calculations. Ann ICRP.

[27] Westrom S, Generalov R, Bonsdorff TB, Larsen RH (2017). Preparation of Pb-212-labeled monoclonal antibody using a novel Ra-224-based generator solution. Nucl Med Biol.

[28] https://radioisotopes.pnnl.gov/isotopes/thorium-227.stm. Accessed 4 May 2018.

